# External quality assurance as a revalidation method for pathologists in pediatric histopathology: Comparison of four international programs

**DOI:** 10.1186/1472-6890-8-11

**Published:** 2008-11-12

**Authors:** Consolato Sergi, Gregor Mikuz

**Affiliations:** 1 Institute of Pathology, Medical University Innsbruck, Austria

## Abstract

**Aim:**

External quality assurance (EQA) is an extremely valuable resource for clinical pathologists to maintain high standards, improve diagnostic skills, and possibly revalidate medical license. The aim of this study was to participate in and compare four international slide survey programs (UK, IAP-Germany, USA-Canada, Australasia) in pediatric histopathology for clinical pathologists with the aim to use it as a revalidation method.

**Methods:**

The following parameters were evaluated: number of circulations per year, number of slides, membership requirement, proof of significant pediatric pathology work, open to overseas participants, laboratory accreditation, issue of continuing professional development certificates and credits, slides discussion meeting, use of digital images, substandard performance letter, and anonymity of responses.

**Results:**

The UK scheme, which has sampling procedure over several time frames (2 circulations/year, 30 slides), partial confidentiality, and multiple sources of data and assessors, can be used as a model for revalidation. The US-Canadian and Australasian schemes only partially fulfill the revalidation requirements. The IAP scheme appears to be essentially an educational program and may be unsuitable for revalidation.

**Conclusion:**

The purposes and programs of EQA schemes vary worldwide. In order for it to be used for revalidation, it is advisable that EQA schemes are immediately unified.

## Background

Clinical Governance is a term, originally used in the National Health Service (NHS) of the United Kingdom (UK), to describe a systematic approach to maintain and improve the quality of patient care. It constitutes an official framework through which the NHS is accountable for the ongoing improvement of service quality safeguarding high standards of care and creating an environment focused on clinical excellence. Where communication failure is the most likely cause for medical errors, the decrease of professional skills may contribute to fatalities. Indeed, medical errors are not usually the result of the failure of particular providers, but are often systems-related and not attributable to individual negligence or misconduct.

Pediatric Pathology embraces two areas of pathology: 1. Specialist organ and system pathology, notably the study of surgical and oncological diseases, but also including the causes, nature and processes of disease and other forms of illness or abnormal conditions of the fetus, newborn, infant and child; and 2. Post mortem examination of the fetus, newborn, infant, and child [1]. Pediatric clinical services rely on safe pediatric pathology services and several initiatives can be employed to keep high the standards of care. In 1999, Quality System Essentials were introduced to laboratory practice by the National Committee for Clinical Laboratory Standards (now Clinical Laboratory Standards Institute [CLSI]) identifying 10 or more major laboratory activities that are important components of a laboratory quality program [2,3]. The essentials include equipment, process improvement, safety and personnel development among others. All of these essentials were developed to ensure that data reported from the laboratory are as accurate as possible and serve the needs of patients and clinicians. An important component in the control of any laboratory procedure is the participation in an external quality assurance (EQA) or proficiency testing program to demonstrate that the method will give the correct result with an unknown random specimen. However, quality assurance in a clinical laboratory also depends on 'safe' pathologists. Thus, EQA may be a fundamental part of the continuing professional development (CPD) of health care professionals [4]. This aspect is recognized internationally and there is a move from continuing medical education (or clinical update) to continuing professional development, including medical, managerial, social, and personal skills.

New compulsory policies for re-validation of doctors in specific areas are currently topical worldwide [5]. If appraisal is a formative and developmental process intended to identify development needs and not performance management, then revalidation is undeniably an episodic process to show capability to practise to the professional regulator (e.g. the General Medical Council in the UK). Thus, revalidation is an assessment that requires a summative judgment (pass or fail). Postgraduate assessment validates doctors for specialist practice initially by allowing them to be entered onto the specialist register, whilst revalidation is the affirmation of continuing fitness to practise and therefore must relate to compliance with defined competencies. The usual academic criteria for such an assessment process include: clear-cut standards, the possible involvement of the public in judging assessments, the use of multiple sources of data and assessors, and sampling procedures carried out over several time frames rather than at a single point [6].

The aim of EQA in pathology is to maintain good running standard operating procedures and to improve the performance of all sub-specialties in order to ensure that patients have access to a high quality service wherever they live. In some countries, laboratories must provide documentation of success in EQA to maintain accreditation or licensure and a number of programs have been developed with assistance from professional bodies together with significant input from accreditation agencies and institutions for improving medical activities [7,8]. In this study, we compare four slide survey programs from four geographical regions (UK, Germany, USA-Canada, and Australasia) with regard to EQA in pathology for pediatric pathologists in the setting of professional development. We discuss the possibility of using these surveys as a method for medical licensure revalidation.

## Methods

Four international tissue slide survey programs the **Bri**tish **P**aediatric **P**athology **A**ssociation (BRIPPA) from the UK , the Royal College of Australasian Pathologists from Australia and South-East Asia – quality assurance programs (RCPA AP QAP) , the North-American Society of Pediatric Pathology (SPP) including US and Canadian subgroups , and the International Academy of Pathology (IAP) – German Division including German speaking countries – Germany, Switzerland, Austria, Lichtenstein, Luxembourg and South-Tyrolean region of North-Italy)  were used in this study. To take into account the different objectives and context of each scheme we evaluated the different EQA programs using the following parameters: circulations per year, number of slides per year, requirement of membership to the regulatory body organizing the EQA, proof of significant pediatric pathology work, open to overseas participants, Clinical Pathology Accreditation Ltd. (CPA) accreditation in the UK or similar accreditation bodies in other countries, issue of CME certificates and CPD or continuing medical evaluation (CME) credits, forthcoming meetings for slides discussion, fixed organizer, annual report issue, improvement changes meeting discussion, digital images option, substandard performance letter, minimum levels of participation, and coding preserving the anonymity of participants. In addition, we also evaluated the participant's option to decide whether some cases are inappropriate for EQA purposes, that some cases require expert opinion or there is either insufficient information or poor quality staining of the glass slides. To assess an EQA program as a revalidation method we studied these surveys from the viewpoint of clear-cut standards, the possible involvement of the public in judging assessments (confidentiality), the use of multiple sources of data and assessors (personal participation), and type of sampling procedures (number of circulations, number of slides per year).

## Results

The EQA scheme for BRIPPA has full CPA accreditation, although re-accreditation according to new standards is due [9]. The last two circulations, i.e. circulation W and circulation X, registered 51 and 52 participants, respectively. The percentage of response was 68.2% for circulation W and 78.8% for circulation X. The Pediatric Histopathology EQA Scheme aims to provide a platform suitable for both pediatric/perinatal pathologists and general pathologists with responsibilities for pediatric pathology. In the UK scheme there is a full range of pediatric surgical material, autopsy material, and placenta. There are two circulations per year in March and October. It is regularly emphasized that the cases are intended to reflect routine practice as far as is possible within the constraints of an EQA scheme. The UK scheme is run by the BRIPPA Committee, who supports the organizer, and is subject to scrutiny by the Royal College of Pathologists EQA Steering Committee and by the National Quality Assurance Advisory Panel (NQAAP) on Histopathology. The participation is voluntary and the scheme is open to both UK and overseas pathologists, provided they work or have worked, at least occasionally, in the UK. In each circulation there are 15 cases that are selected by the organizer on the basis of the clinical history alone. It is advised that any relevant clinical information existing when the original report was dispatched is provided and that this material should be made available to all participants. Each participant is given a numeric code, which is entered on the return forms to permit a personal statistical analysis of results. Reports giving the range and popularity of the diagnoses are sent to all participants, along with the comments and the original diagnoses. Histograms or other statistical figures are used to give the distribution of the accumulated scores for of each participant. This allows the participant to see confidentially how his/her performance compares with his/her peers. The computer program which runs this system has been previously described [10]. The program allows calculation of the degree of correlation between the personal diagnosis and that formulated by the consensus of the group. The numerical code also permits anonymity and confidentiality and is known only to a part-time secretary employed on the scheme. There is an annual meeting at which cases are presented and discussed and personal scores are calculated after the suitability of each case for EQA purposes has been discussed at such a meeting. There is a definition of a "persistent sub-standard performance" which is defined as any participant whose score remains in the bottom 2.5% for two circulations. Anonymity is broken if the chairman can identify that standards of care are jeopardized. In the event of a pathologist making diagnoses which are markedly at variance with the consensus, the feedback system should make that pathologist aware of the position. A bottom line of 2.5% of participants has been proposed [11]. A number of procedures have also been proposed where the Chairman of the Histopathology Advisory Panel will be informed if he/she again scores in the bottom 2.5% of the ranked order in two out of the next three circulations [12]. It is advisable to start an investigation without breaking confidentiality, which seeks explanations rather than taking punitive action. Such procedures have been approved by the Council of the RCPath and are described in more detail on the College website [13]. The organization of the scheme requires the maintenance of a computer, postage, packing, and photocopying costs and a part-time secretary. Thus, a number of positive aspects are present in this scheme if compared with the other three (Table 1). Concerning the evaluation of the BRIPPA EQA as a revalidation method it should be indicated that such a scheme has partial confidentiality, multiple sources of data and assessors, and a sampling procedure over several time frames, fulfilling almost all criteria necessary for a revalidation method.

**Table 1:** Factors used in the evaluation of histopathology EQA schemes

	**BRIPPA**	**RCPA AP QAP**	**SPP**	**IAP-GD**	***Comprehensive *EQA^6^**
**No. of Circulations/year**	2	1	3	1	2
**No. slides/year**	30	10	15	50 ^5^	30
**Membership Required**^1^	Yes	No	Yes	Yes	Yes
**Pediatric Pathology Required**	Yes	No	Yes	No	No
**Open to Overseas participants**	Yes ^3^	Yes	Yes	Yes	Yes
**CPA accreditation (UK or similar accrediting bodies in other countries)**	Yes	Yes	Yes	Yes	Yes
**CPD/CME certificates**	Yes	Yes	Yes	Yes	Yes
**CPD/CME credits**	10	10	15	10	15
**Slides Discussion Meeting**	Yes	No	No	Yes	Yes
**Fixed Organizer**	Yes	Yes	Yes	Yes	Yes
**Annual Report Issue**	Yes	Yes	Yes	No	Yes
**Improvement/Changes Meeting Discussion**	Yes	Yes ^4^	Yes	No	Yes
**Digital Images option**	No	Yes	No	No	Yes
**Substandard Performance Letter**	Yes	No	No	No	Yes
**Minimum Levels of Participation**	Yes	No	No	No	Yes
**Anonymous Responses**^2^	Yes	No	No	No	Yes
**Participant's option to reject EQA cases where inappropriate for the EQA requiring actually expert opinion**	Yes	Yes	No	No	Yes
**Participant's option to reject EQA cases due to lack of sufficient information**	Yes	Yes	No	No	Yes
**Participant's option to reject EQA cases due to poor staining quality**	Yes	Yes	No	No	Yes

Notes: ^1 ^The membership of the regulatory body (BRIPPA, RCPA, SPP, IAP) organizing the EQA is a requirement to participate in the histopathology survey; ^2 ^the parameter 'anonymous responses' indicates that a coding is used to preserve the anonymity of the participants (It also means indirectly that explicit permission from the participant is required before data may be shared with local management, regional QA officers, accrediting bodies and suppliers of equipment and reagents); ^3 ^with limitations (membership for pathologists is usually restricted to those with a regular commitment to work, usually locum work, in the United Kingdom); ^4 ^with service online; ^5 ^permanent set of stained sections (probably unsuitable for revalidation); ^6 ^The comprehensive EQA is considered as major acceptance and author recommendation among the different EQAs. Neither EQA organizations nor the Medical University of Innsbruck are responsible for these conclusions.

BRIPPA: British Paediatric Pathology Association,

RCPA AP QAP: Royal College of Pathologists of Australasia Anatomical Pathology Quality Assurance Programme,

SPP: Society for Pediatric Pathology

IAP: International Academy of Pathology

CPA: Clinical Pathology Accreditation Ltd.

The EQA scheme of the Society of Pediatric Pathology runs acceptably. In particular, the slide survey program of the Society of Pediatric Pathology (USA) contains an important educational component using three single choice questions ("best of four") in addition to the field "Your diagnosis". In this sense the credits gained by the survey program are corroborated by active research of the participant in the medical literature and the educational value of the survey is perceptibly increased. Although the use of a multiple choice question is a valuable and probably stimulating aspect of continuing medical education, it remains of educational value, because it is so far from routine practice as to be irrelevant to EQA. A digital slide survey program has recently been started in late 2007, but a full evaluation is lacking.

The slide box of the German Division of the International Academy of Pathology allows pathologists working in a German-speaking country to familiarize themselves with pediatric pathology entities. A feedback form is included, but it is not compulsory. A standard course (about once a year) is offered explaining the diagnoses and differential diagnoses as well as inserting additional comments or molecular pathology notes. Digitalization of data is in progress. At present four modules are offered for pediatric pathologists and general pathologists with a significant workload in pediatric pathology. No substandard performance letter is issued.

The specialist module "Pediatric Pathology" of the Royal College of Pathologists of Australasia, Section of Anatomical Pathology, Quality Assurance Programme (RCPA AP QAP) is accredited by the National Association of Testing Authorities, Australia, and complies with the Requirements of ILAC G13 [14]. The 2007 survey was distributed to 75 participants in a range of Australian and international laboratories: Australia 63%, New Zealand 12%, Malaysia 12%, Other (Austria, Fiji, New Caledonia, Saudi Arabia, Singapore, South Africa, Sweden, United Arab Emirates) 13%. A major change for 2006 was the increase of cases for each specialist module, now including a total of ten slides per year. Initially slide sets and additional digital files for virtual microscopy are provided. In 2005 the Royal College of Pathologists Australasia acquired the Aperio ScanScope^® ^system [15]. This system, which is in addition to existing virtual microscopy equipment, was funded under a Commonwealth government contract and is fully operational at the RCPA QAP office. The virtual microscopy equipment is able to scan diagnostic slides and create digital images that can be magnified in a similar manner to light microscopy. The equipment contains special features including a particular system of file compression that allows highest resolution of histological glass slides and the files are accessible through a free ware viewing program for personal computers. In response to a questionnaire sent to RCPA AP QAP participants, it was found that about two thirds of participants were able to download the software and open the digital files, although only one out of two respondents had personal access to a personal computer with the minimum requirements for viewing the scanned slides. About one third of the participants upgraded a personal computer during the following 12 months to establish all of the minimum requirements and a further one third was able to upgrade to all of the ideal requirements for viewing the images. Three quarters of the respondents made a comment about the use of virtual microscope images for EQA purposes. Of these comments one third were positive, while the negative responses were mostly associated with the IT difficulties found at workplace, such as firewalls or the lack of DVD ROM drives. In the RCPA AP QAP module preferred diagnoses can be submitted electronically (website) or by fax or post. One of the most important achievements is the acceptance of website submitting and interim results are available on the RCPA AP QAP website immediately after the closing date. Assessment criteria include five categories for the preferred diagnosis classified against the target diagnosis. The diagnosis is classified concordant, if the preferred diagnosis is essentially/substantially identical with the target diagnosis; minor discordant, if the preferred diagnosis has one or more minor differences from the target diagnosis; discordant, if the preferred diagnosis is substantially different from the target diagnosis, differential diagnoses only, if only a number of differential diagnoses are reported, and unable to be assessed, if the submission was late, illegible or unable to be interpreted, including submissions in the form of a clinical report, a fax instead of a web submission or those with no text in the "preferred diagnosis" field. Participation as an individual in any of the diagnostic modules is recognized as a CPD activity by the Board of Education of the Royal College of Pathologists of Australasia.

If we consider all four programs together, we recommend that an ideal quality assurance scheme should have, on average, two circulations per year for a total of at least 30 slides per year, be addressed to pathologists with a substantial pediatric pathology workload, are accredited, provide CPD credits and certificates, and have regular slides discussion meetings as well as scheme improvement meetings, and lastly issue an annual report (Table 1).

## Discussion

Clinical governance is one of the most frequent terms encountered in health care management, in both the national health services and private healthcare systems. It describes a systematic approach maintaining and regularly improving the quality of patient care while safeguarding high standards of care through creation of an environment in which excellence in clinical care will flourish. The elements of clinical governance advance health care professional education, clinical audit, clinical effectiveness, risk management, good professional practice-based research and development, confidential-based honesty procedures. In this study, we delineate the characteristics of an EQA scheme that should be taken into consideration when establishing a national or local EQA scheme. We consider that if a quality assurance scheme is to be suitable as revalidation method it should have two circulations per year, requirement for membership or a substantial pediatric pathology workload. It should be open to overseas participants, have CPA accreditation, provide CPD credits and certificates, and have regular slides discussion and scheme improvement meetings and an annual report. Internal audits and a 'black box' of tissue slides are important, but external proficiency testing is advocated as an independent means for continuing professional development and to support laboratory accreditation. Indeed, laboratory services are a central core in the diagnostic procedures of a general hospital [16]. The "Pediatric Pathology" module of slide survey programs is targeted for pediatric pathologists, general pathologists who have pediatric cases in their diagnostic routine, pediatric pathology fellows and pathology residents.

The concept of EQA programs relies on the fact that participants should view cases without consulting colleagues before submission to the program office. Uniformity of standards to set up a quality assurance program at national level is an important task. The use of physician specialization to assess the quality of care provided by individual physicians represents a structural approach to measuring quality. In the last 10 years there has been an enormous change in revalidation in the United States. This is because physician specialization as represented by board certification may be an unreliable measure of the quality of a physician's performance over time, unless his or her knowledge and skills in a specialty area are periodically updated or assessed.

In such a way, there is no guarantee that physicians have maintained the same level of skills and knowledge they demonstrated for their initial certification without a revalidation process. Thus, the search for a practicable revalidation method is very valuable. According to our data and considerations, the UK scheme has an educational component largely as a by-product of participation, but it can easily be used as a form for medical revalidation. This is because it possesses a partial confidentiality, contains multiple sources of data and assessors, and has a sampling procedure over several time frames rather than at a single point. The German scheme would appear to be essentially that of an educational program and possibly the North American scheme too. The Australian scheme seems to have both educational and revalidation components, but the number of slides is low and there is only one circulation per year that we consider insufficient as a revalidation method.

In assessing EQA schemes one aspect that has to be emphasized is the 'consensus answer'. The EQA utilizes the consensus answer as the correct answer which may not necessarily be the same as an 'expert' answer. Inter-observer reliability (consistency) may be reassuring, but there is a risk of a group response that can become very unpredictable. [17] The participants may all agree, but the correct or best answer may not be made as all of the participants are wrong. Similarly, an expert opinion is not necessarily correct. It is well recognized in pathology that there are instances when pathologists cannot agree as to the correct answer even on cases that are not unusual or rarities. Disagreement between experts is to be expected and should not necessarily give rise to concern over their competence [18]. Consequently, the EQA, rather than measuring expertise, is measuring a minimum common level of attainment (i.e. conformity to consensus). Therefore how the consensus should be assessed? There are several forms of consensus used today. The promise of achieving a consensus creates the impression that one group may be able to operate without conflict. Consensus' philosophy fosters an atmosphere that encourages the expression of different opinions and even conflict, and then provides a process that may lead to a resolution in a creative and very supportive environment. It recognizes that everyone has a contribution to make, and that all views are encouraged, but it may take several meetings to work towards a decision and at the end of the process it is not uncommon for some people to continue to disagree with the final decision. The Delphi Technique and Nominal Group Technique are two well-recognized consensus-formation methodologies specifically designed to combine judgments from a group of experts [19,20]. The Delphi Technique utilizes a series of well-defined questionnaire-based surveys, whereas Nominal Group Technique is a structured face-to-face meeting designed to facilitate consensus. Consensus-formation techniques require that each step builds on the results of the previous steps. In the majority of the EQA survey programs for pathologists the correct diagnosis is not indicated as 'expert judgment' but conversely a 'democratic' consensus is sought by judgment of the majority of participants and discussion in the case of conflicting results. In some situations, a consensus cannot be reached and the controversial tissue slide is excluded from the final EQA performance. The rejection of cases not reaching 80% agreement between participants can be criticized, as it is artificial, but it is a compromise [21]. As a consequence of rejecting such cases, the distribution of the score profile becomes skewed.

Another aspect at the base of some controversial issues in EQA in pathology may be the lack of uniform guidelines that are valid worldwide. Thus, our study first tries to compare four international survey programs, emphasizing the need for harmonization. However, lack of harmonization also relies on the different interpretation of some pathology. An example is the diagnosis of chronic intestinal pseudo-obstruction in children. The diagnosis of intestinal neuronal dysplasia as an isolated entity is indeed very controversial. Intestinal neuronal dysplasia is characterized by hyperplasia of the submucosal and myenteric plexuses and the isolated form has been described in the distal colon and rectum, and its clinical presentation, with constipation and intestinal obstruction, can mimic that of Hirschsprung disease or aganglionosis [22,23]. During the years since the entity was first described, the criteria have also been modified. However, in many countries, mostly of Anglo-Saxon origin, the current view is that isolated intestinal neuronal dysplasia is seen in a variety of clinical settings and is more a descriptive entity than a specific disease requiring surgical intervention [24].

It is difficult to identify the minimal number of slides that needs to be assessed for consideration as a revalidation method. We initially considered the number of cases of pediatric and placental pathology in a tertiary academic center. The number of cases of pediatric and placental pathology was found to be approximately 1500 in many institutions (personal communication) over the years. Subsequently, we considered the percentage of 'permitted wrong diagnoses' in the diagnostic routine identified in the English literature. The results of studies concerned with error rates in histopathology vary widely; no serious errors, [25] 0.26%, [26] up to 1.2% of histopathological reports [27]) but these were performed in academic or teaching institutions. In reality, the percentage is quite variable when considering sub-specialistic, inter-individual and intra-individual variability studies [28]. It has also been suggested in the USA that false negative rates of 5–10% may be an admirable goal in cytopathology, and rates below 15–20% are a possible standard [29]. We allowed 2% in consideration of typographical errors, and hypothesized that a valuable number of EQA diagnoses could be 30 (2% of 1500 diagnoses), keeping in mind an artificial assumption that the highest number of wrong diagnoses can be equivalent to the number of histological glass slides that run in a annual EQA program. Thus, we propose that 30 should be the minimal number of slides per year assessed in an EQA program by a specialist pediatric pathologist. In this sense, the UK scheme with 30 slides per year might be considered as a standard and may serve better as a revalidation method.

Health services are awash with data, but safety is a category that needs to be continuously improved for health services. IT resources appear to be the greatest barrier to obtaining access to virtual images. This can be overcome if hospitals or government upgrade workstations to have compatible IT systems and allow access for quality assurance purposes. Smooth running quality assurance programs can improve this relationship and strengthen the link with the clinicians. Modernizing the pathology laboratory commences with virtual microscopy and services will provide a remarkable wealth for the child's growth in the 3^rd ^millennium. Image digitalization is a new tool to use for biopsy specimens that cannot be cut in 40–60 slides for all participants. Indubitably, digital imaging with virtual microscopy will be more closely linked to practice in the future. To date, glass slides mimic the routine practice worldwide. Thus, digital images for EQA are a compromise offering the possibility to extend the range of cases to include small specimens, such as endoscopic and fine needle biopsies. Another possibility is to manage the use of glass slides from small biopsies through a postal system, circulating slides sequentially between participants. In the recent UKNEQAS Meeting in Glasgow it was stated that the EQA remains an extremely valuable resource for clinical pathologists and needs a well-organized, rapid and manageable system to run efficiently [31].

It seems evident that EQA drives standardization and there are many examples supporting this fact, including those where peer-driven changes are influenced by EQA findings [31]. One aspect that was not considered in this study was the difficulty of the histological glass slides utilized in the circulations. However, in our opinion, there was no significant disparity in the difficulty of diagnosis because some recommendations have been proposed and followed in all four EQA schemes examined. In particular, the diagnosis should be made using the hematoxylin and eosin stained slide and no immunohistochemistry should be needed to arrive to the diagnosis. Lymph node cases usually represent a frequent source of difficulty and specific details have to be provided in submitting such cases. Thus, the method for selecting cases has a crucial impact on whether the cases are more difficult (and hence more educational) or more representative of the routine workload (and therefore more relevant to performance surveillance). Cases should be contributed by all participants in rotation, following agreed guidelines. Extremely 'simple' cases should be avoided, to be determined at meetings of the participants, but bizarre cases and case-report material remain inappropriate. The number of cases circulated must be sufficient to give reasonable confidence that serious sub-standard performance will be promptly identified.

Inappropriate tissue slides, limited number of circulations, lack of secretarial support, and low number of participants may jeopardize the EQA as a revalidation method. The importance is to establish a quality management system, allocate privileged time for it and ensure a reasonable cost load for healthcare organizations. It is important to specify well defined implications for schemes and participants. Further, we believe that a periodically reviewed and updated quality policies manual in addition to continuing audit of performance should be standard in every histopathology department. The association with a CPA accredited laboratory (contractual agreement) should be considered. If an EQA scheme is not run in a department, it is recommended that the department is not given accreditation.

Business and healthcare organizations regularly use the process of benchmarking to learn how others address policy issues and solve problems. The improvement of diagnostic skills in pathology is of paramount importance and the interest in programs that provide external proficiency testing, quality assessment and appropriate education programs to public and private laboratories of pathology and laboratory medicine is growing rapidly. There are some associations and companies supplying both quality assurance programs and supporting services for the benefit of pathology laboratories and personnel working within the pathology environment. The substantial advantage of internal audit and slide survey is intrinsically present in these programs intended to continually improve pathology services for the well being of communities. There is no direct evidence to support the validity of histopathology EQA, but there are various strands of indirect evidence that can be drawn together to underpin validity. Indirect evidence to support validity includes the response process used in the EQA. This reflects actual working practice undertaken by pathologists. EQA started originally as a 'hobby' for pathologist participants, but now it has acquired or is acquiring a central role as part of continuing professional development. It may be used as a revalidation method, because it represents clear of medical qualification, its' results may be given to the public, it may contain multiple sources of data and assessors and it represents a sampling procedure over several time frames rather than at a single point. However, the purposes and programs of EQA schemes can be different worldwide and in consideration of its possible use as revalidation method, it is strongly advisable that all EQA programs are unified as soon as possible.

## Authors' contributions

CS conceived of the study, participated in its design and coordination, participated actively to all four EQA programs, analyzed all data and wrote the manuscript. GM reviewed critically the manuscript.

## Pre-publication history

The pre-publication history for this paper can be accessed here:


